# Effect of Waterlogging-Induced Autophagy on Programmed Cell Death in *Arabidopsis* Roots

**DOI:** 10.3389/fpls.2019.00468

**Published:** 2019-04-11

**Authors:** Bin Guan, Ze Lin, Dongcheng Liu, Chengyang Li, Zhuqing Zhou, Fangzhu Mei, Jiwei Li, Xiangyi Deng

**Affiliations:** ^1^Laboratory of Cell Biology, College of Life Science and Technology, Huazhong Agricultural University, Wuhan, China; ^2^College of Plant Sciences and Technology, Huazhong Agricultural University, Wuhan, China; ^3^College of Food and Biological Science and Technology, Wuhan Institute of Design and Sciences, Wuhan, China

**Keywords:** reactive oxygen species, respiratory burst oxidase homolog, autophagy, programmed cell death, waterlogging

## Abstract

Autophagy, a highly conserved process in eukaryotes that involves vacuolar degradation of intracellular components and decomposition of damaged or toxic constituents, is induced by endogenous reactive oxygen species (ROS) accumulation, endoplasmic reticulum stress, and other factors. In plants, the role of autophagy in the induction of programmed cell death (PCD) is still unclear. Here, we show that ROS contribute to the regulation of PCD during waterlogging (which results in oxygen depletion) via autophagy. In wild-type roots, waterlogging induces the transcription of hypoxia-responsive genes and respiratory burst oxidase homolog (RBOH)-mediated ROS production. It also altered the transcription level of alternative oxidase1a and the activity level of antioxidant enzymes. Moreover, waterlogging increased the transcription levels of autophagy-related (*ATG*) genes and the number of autophagosomes. Autophagy first occurred in the root stele, and then autophagosomes appeared at other locations in the root. In *rboh* mutants, upregulation of autophagosomes was less pronounced than in the wild type upon waterlogging. However, the accumulation of ROS and the level of cell death in the roots of *atg* mutants were higher than those in the wild type after waterlogging. In conclusion, our results suggest that autophagy induced in *Arabidopsis* roots during waterlogging has an attenuating effect on PCD in the roots.

## HIGHLIGHTS

- This study examined the impact of waterlogging-induced autophagy on programmed cell death in *Arabidopsis* roots.

## Introduction

Flooding, which results in soil waterlogging, and in many situations complete submergence, is a major issue for plant survival in many regions of the world. When waterlogging occurs, restricted gas exchange between the soil and atmosphere and altered respiratory processes of soil organisms contribute to hypoxia and anoxia (Mühlenbock et al., 2007). Under hypoxic or anoxic conditions, ethanol produced by anaerobic respiration and reactive oxygen species (ROS) leakage from mitochondria affect root growth and function. To cope with a shortage of oxygen, some gramineous plants form lysigenous aerenchyma in their roots as a consequence of cell death of cortical cells (Jackson and Armstrong, 1999; Evans, 2003).

Previous studies of *Arabidopsis thaliana* have shown that the expression of genes encoding enzymes related to glucose metabolism, glycolysis, and fermentation increases in response to hypoxia (Liu F. et al., 2005; Loreti et al., 2005; van Dongen et al., 2009). Under hypoxic conditions, the transition of plants from aerobic respiration to lactic acid fermentation increases the level of expression of the lactate dehydrogenase (*LDH*) gene (Kreuzwieser et al., 2009). Because the accumulation of lactic acid leads to the acidification of the cytoplasm, the activity of LDH is reduced, allowing alcohol fermentation (Kreuzwieser and Rennenberg, 2014). The activities of enzymes required for alcohol fermentation, which include pyruvate decarboxylase (PDC) and alcohol dehydrogenase (ADH), are higher in waterlogged roots (Atkinson et al., 2008). Sucrose synthase (SUS), which participates in sucrose metabolism, is of great importance to surviving hypoxia (Santaniello et al., 2014). A specific subfamily of transcription factors, Group VII ethylene response factors (ERF-VIIs), plays important roles in regulating the activation of fermentation-related genes (Bailey-Serres and Voesenek, 2008). In plants, ERF-VII genes have been found to play a role in the oxygen-sensing mechanism through the N-end rule pathway of proteolysis (Gibbs et al., 2011; Licausi et al., 2011). Under hypoxia, ERF-VIIs are released and activate the expression of hypoxia-responsive genes, including the fermentative genes *PDC1* and *ADH* (Gasch et al., 2016).

Well-known ROS molecules in plants include ozone, singlet oxygen, the superoxide anion, hydrogen peroxide (H_2_O_2_), and hydroxyl radicals. ROS signaling, especially that of H_2_O_2_, plays a key role in plant adaptation to low-oxygen conditions (Dat et al., 2004; Bailey-Serres and Voesenek, 2008; Sauter, 2013; Pucciariello and Perata, 2017). H_2_O_2_ is produced by the double electron reduction of molecular oxygen catalyzed by the respiratory burst oxidase homologs (RBOHs), which is a plant homolog of mammalian nicotinamide adenine dinucleotide phosphate (NADPH) oxidase in the plasma membrane (Torres et al., 1998). The *Arabidopsis* genome has 10 *RBOH* genes, and each homolog has a specific role in different biological processes (Foreman et al., 2003). The expression of the *RBOH* gene contributes to the production and signaling of *RBOH*-dependent ROS in the immune response of *A. thaliana* (Morales et al., 2016). However, the functions of RBOHs under hypoxic or anoxic conditions are poorly understood.

In the plant mitochondrial electron transport chain (ETC), there are two terminal oxidases, cytochrome oxidase and alternative oxidase (AOX). AOX is an integral protein of the inner mitochondrial membrane and catalyses the alternative respiratory pathway (Feng et al., 2013). Under environmental stresses, electrons produced by the respiratory oxidation of NADPH can flow through the alternative respiratory pathway instead of the usual cytochrome respiratory pathway in higher plants. This can limit the excessive production of ROS and nitric oxide and maintain the redox balance of plant cells (Maxwell et al., 1999; Giraud et al., 2008; Smith et al., 2009; Cvetkovska and Vanlerberghe, 2012). Enzymatic mechanisms to scavenge overproduced ROS in plants include superoxide dismutase (SOD), ascorbate peroxidase (APX), and catalase (CAT). The main function of SOD is dismutation of superoxide to H_2_O_2_, which is detoxified to H_2_O and O_2_ by APX and CAT (Apel and Hirt, 2004).

Autophagy is a highly conserved degradation process that transfers intracellular components to vacuoles or lysosomes under environmental stress or at certain developmental stages (Klionsky and Ohsumi, 1999; Klionsky, 2005, 2007). In plants, two major autophagic pathways have been described, microautophagy and macroautophagy (hereafter, autophagy) (Bassham et al., 2006). During autophagy, damaged or toxic constituents are engulfed in a double membrane vesicle called an autophagosome, which fuses with the vacuole to deliver the autophagic body into the vacuolar lumen for further break-down (Li and Vierstra, 2012; Liu and Bassham, 2012). Autophagy is active at very low levels during plant growth and development but is highly inducible in response to a variety of abiotic stresses, such as oxidative stress, salt stress, osmotic stress, and heat stress (Zhou et al., 2014). Autophagy-defective (*atg*) mutants or transgenic plants are more hypersensitive to ROS, ethanol, and submergence conditions than wild-type plants (Chen et al., 2015). In mammals, ROS is an important stimulator of autophagy induction, whereas autophagy reduces the accumulation of ROS through the removal of damaged organelles (Li et al., 2015). The interaction between ROS and autophagy has also been reported in plants recently. Autophagy is induced after treatment of *Arabidopsis* plants with H_2_O_2_ or ROS-producing agent methylviologen (MV). After treatment with MV, RNAi-*ATG18a* seedlings accumulated more oxidized proteins and were more sensitive to oxidative stress (Xiong et al., 2007).

Programmed cell death (PCD) is a planned, step-by-step, active suicide process that selectively eliminates unnecessary or damaged cells in eukaryotes (Gadjev et al., 2008). PCD is a genetically controlled form of cell death, both in the course of development and in response to osmotic, thermal, and oxidative stresses and in defenses against pathogens. Like animal cell apoptosis, PCD in plants shares common morphological and biochemical features, including cell contraction, cytoplasmic contraction, cytochrome *c* release from mitochondria, excessive production of ROS, chromatin condensation and aggregation, and DNA fragmentation. Recent studies have shown that multifarious PCD processes are accompanied by autophagy (Liu Y. et al., 2005). An interesting view has developed in recent issues of *Trends in Plant Science*, autophagy plays a unique role in the initiation, execution of, and protection against cell death (Minina et al., 2014), suggesting that the morphological features and mechanisms of autophagy may be different in living cells and dying cells. Therefore, we plan to perform further work to determine the exact role of autophagy in the induction of PCD in plant cells.

Recently, identification of autophagy-related (*ATG*) genes and research on the autophagy pathway have made considerable progress (Bassham et al., 2006; Liu and Bassham, 2012). Several studies have demonstrated the pro-survival role of autophagy in plant cells under environmental stresses (Doelling et al., 2002; Xiong et al., 2007; Shin et al., 2009) including submergence (Chen et al., 2015). We further investigated the impact of waterlogging-induced autophagy on PCD in *Arabidopsis* roots. Here, we report that ROS**-**dependent autophagy has an attenuating effect on PCD in waterlogged *Arabidopsis* roots.

## Materials and Methods

### Plant Materials, Growth Condition, and Treatments

The strains of *A. thaliana* used in this study were all Columbia-0 (Col-0) ecotype. *A. thaliana* mutants *atg2* (SALK_076727), *atg5* (SAIL_129_B07), *atg7* (SAIL_11_H07), *atg10* (SALK_084434C), and *rboh* (*rbohd*, CS9555; *rbohf*, CS9557 and *rbohdf*, CS9558) were obtained from the Arabidopsis Information Resources Centre. Seeds of plants expressing *35Spro:GFP-ATG8e* and *ATG8epro:GUS* (Chen et al., 2015) were kindly provided by Prof. Shi Xiao (Sun Yat-sen University). All *Arabidopsis* seeds were surface sterilized in 20% bleach [1% (v/v) NaClO] and 0.01% Triton X-100 for 12 min, washed five times with sterile water, and stored at 4°C for 3 days. Then, the seeds were sown on plates containing half-strength MS medium with 1% sucrose and 0.8% (w/v) agar. The plates were vertically placed in growth chambers with a 16-h-light/8-h-dark cycle at 22°C.

All waterlogging treatments were performed in three independent biological replicates. Waterlogging treatment was carried out as described previously, with minor modifications (Wang et al., 2016). Briefly, 1-week-old seedlings were transferred to pots containing a stagnant liquid MS medium with 1% sucrose. The stagnant solution contained 0.2% (w/v) dissolved agar to prevent convective movement and was deoxygenated (dissolved O_2_, <0.5 mg/L) by flushing with N_2_ gas (Wiengweera et al., 1997; Yamauchi et al., 2017). The plant roots were completely immersed in stagnant solution, and leaves protruded 0.5 cm above the surface of the solution. The plants were placed in chambers at 22°C under a 16-h photoperiod. In order to calculate root length and survival rates after waterlogging, more than 20 plants of each genotype were waterlogged. Survival rates were based on the ability to produce new leaves and continue to grow.

### Detection of Autophagy

Acid dyes, i.e., monodansylcadaverine (MDC) and GFP-ATG8 fusion proteins, are often used to detect autophagy in plants (Contento et al., 2005). Seedlings (1 weeks old) expressing *GFP-ATG8e* fusion protein were waterlogged with stagnant solution for the indicated times. GFP fluorescence was observed and photographed using a Leica SP8 laser scanning confocal microscope (Leica, Germany), and the excitation and emission wavelengths were 488 and 507 nm, respectively.

Monodansylcadaverine staining was carried out as described previously (Contento et al., 2005). Briefly, seedlings (1 weeks old) were waterlogged for the indicated times and subsequently incubated in 0.05 mM MDC (Sigma) in PBS for 10 min, followed by three washes with PBS at room temperature. Seedling were observed and photographed with a Leica SP8 laser scanning confocal microscope using a DAPI-specific filter. The excitation and emission wavelengths for MDC were 345 and 455 nm, respectively.

Each sample consisted of three independent biological replicates. Three roots per experiment were analyzed microscopically. Five optical sections per root were counted. The thickness of a section is 1 μm. The *GFP-ATG8e*-labeled and MDC staining punctate structures were counted using the Adobe Photoshop software^1^.

### ROS Assays

We used diaminobenzidine (DAB) staining to detect H_2_O_2_ *in situ* as described previously, with minor modifications (Yang et al., 2014). Seedlings (1 weeks old) were incubated in 0.5 mg/mL DAB (Sigma) dissolved in 50 mM Tris–HCl (pH 5.0) for 2 h. The seedlings were rapidly washed three times with water and observed on a differential interference contrast microscope (Nikon 80i Eclipse). The quantitative measurement of staining intensity was carried out using Image pro plus 6.0.

Nitroblue tetrazolium (NBT) staining was performed to detect superoxide anion as described previously (Dunand et al., 2007). Briefly, seedlings (1 weeks old) were steeped in 2 mM NBT in 20 mM phosphate buffer (pH 6.1) for 15 min. The seedlings were transferred to distilled water and observed on a differential interference contrast microscope (Nikon 80i Eclipse). The quantitative measurement of staining intensity was carried out using Image pro plus 6.0.

For 2′,7′-dichlorofluorescin diacetate (DCFH-DA) staining to detect ROS (Soh, 2006; Kalyanaraman et al., 2012), 1-week-old seedlings were incubated at 37°C in 10 μM staining solution for 30 min. The DCFH-DA (sigma) was diluted from a 10 mM stock in ethanol using PBS. The stained seedlings were rapidly washed with PBS three times and were observed on a Leica SP8 laser scanning confocal microscope with an excitation wavelength of 488 nm and an emission wavelength of 525 nm. The quantitative measurement of fluorescence intensity was carried out using Image pro plus 6.0.

The content of H_2_O_2_ was measured as described previously (Patterson et al., 1984). The content of superoxide anion was determined according to hydroxylamine method (Elstner and Heupel, 1976).

### GUS Assays

For the GUS staining assay, 1-week-old seedlings were soaked in GUS assay buffer [0.1 M phosphate buffer (pH 7.0), 5 mM K_4_Fe(CN)_6_, 5 mM K_3_Fe(CN)_6_, 0.1% Triton X-100, and 0.5 mg/mL X-Gluc] at 37°C in the dark for 9 min and were then incubated in 75% ethanol. Seedlings were photographed with a differential interference contrast microscope (Nikon 80i Eclipse).

### Antioxidant Enzymes Extraction and Assays

One-week-old *Arabidopsis* roots (1.0 g) prepared for enzyme activity were homogenized in an extraction solution containing 50 mM Na_2_HPO_4_–NaH_2_PO_4_ buffer, 0.2 mM EDTA, and 2% insoluble polyvinylpyrrolidone. The homogenate was centrifuged at 12,000 rpm for 20 min and the supernatant was used to determine enzyme activity. The activity of SOD, APX, and CAT was determined by using SOD assay kit, APX assay kit, and CAT assay kit purchased from Nanjing Jiancheng Bioengineering Institute according to manufacturer’s directions (Hussain et al., 2016).

### RNA Extraction and Quantitative Real-Time PCR Analysis

Total RNA was extracted from 1-week-old *Arabidopsis* roots using TRIzol reagent (Invitrogen) according to the manufacturer’s instructions. The isolated RNA was reverse transcribed using the PrimeScript RT reagent kit with gDNA Eraser (Takara). Reverse transcription quantitative PCR (qRT-PCR) was performed with SYBR Premix Ex Taq II (Takara) using a StepOne Plus real-time PCR system (Applied Biosystems). The program for qRT-PCR was 95°C for 30 s, 40 cycles of 95°C for 5 s, and 60°C for 30 s. Each reaction had three biological and three technical replicates. *ACTIN2* was used as the internal control and transcription levels were calculated according to the 2^-ΔΔCt^ formula. Information of genes included in this study are listed in Supplementary Table S1. Primers used for qRT-PCR analysis are listed in Supplementary Table S2.

### Transmission Electron Microscopy

The general procedures of preparing conventional samples for transmission electron microscopy (TEM), described previously, were followed with minor modifications (Xu et al., 2013). Briefly, middle areas of root sections (1 mm^3^) were cut from seedlings (1 weeks old) and fixed immediately in 2.0% paraformaldehyde and 2.5% glutaraldehyde in PBS overnight at 4°C. The tissues were rinsed three times using PBS, post-fixed in 1% osmium tetroxide for 4 h, rinsed three times using PBS again, dehydrated in a graded acetone series, and embedded in SPI-PON 812 resin (SPI Supplies). For TEM, the ultrathin sections were contrasted with uranyl acetate and lead citrate and were observed directly under the TEM (Hitachi H-7650, Japan) at 80 kV. For statistical analysis of the autophagic structures, five roots for per experiments and three cells from every root/root tissue were analyzed microscopically.

### Protein Extraction and Western Blot Analysis

Total protein was extracted from 1-week-old *Arabidopsis* roots using an ice-cold RIPA lysis buffer (50 mM Tris, pH 7.4, 150 mM NaCl, 1% Triton X-100, 1% sodium deoxycholate, 0.1% SDS) supplemented with 1 mM phenylmethanesulfonyl fluoride (PMSF; Roche). The homogenates were placed on ice for 30 min and were then centrifuged at 4°C for 15 min at 12,000 rpm. The supernatant was transferred to a new microfuge tube before electrophoresis.

For the immunoblot analysis, total proteins were separated by 15% SDS–PAGE and were then electroblotted to a nitrocellulose membrane (Biosharp). Anti-AOX (agersera), anti-Actin (sigma), anti-ATG8a (Abcam), and anti-GFP (Abcam) antibodies were used in the immunoblotting analysis. Quantification of the protein signal was performed using Image pro plus 6.0 software.

### Cell Death Detection

For phenotypic analysis, 1-week-old *Arabidopsis* roots under normal or waterlogged conditions were stained using 10 μg/mL propidium iodide (PI; Sigma) and were observed on a Leica SP8 laser scanning confocal microscope with an excitation wavelength of 535 nm and an emission wavelength of 615 nm.

For FDA staining, 1-week-old *Arabidopsis* roots under normal or waterlogged conditions were stained with 5 ug/mL Fluorescein diacetate (FDA; Sigma) for 5 min and were washed three times using sterile water. The fluorescence signal was observed on a Leica SP8 laser scanning confocal microscope with an excitation wavelength of 488 nm and an emission wavelength of 525 nm. The quantitative measurement of fluorescence intensity was carried out using Image pro plus 6.0.

For trypan blue staining, 1-week-old seedlings were placed at room temperature in trypan blue staining buffer (0.4% solution in PBS) for 10 min, followed by washing three times using PBS. Seedlings were photographed with a differential interference contrast microscope (Nikon 80i Eclipse).

For DAPI staining, 1-week-old *Arabidopsis* roots under normal or waterlogged conditions were stained in PBS containing 1 μg/mL 4′,6-diamidino-2-phenylindole (DAPI; Sigma) for 30 min and washed three times with PBS. The fluorescence signal was observed on a Leica SP8 laser scanning confocal microscope with an excitation wavelength of 358 nm and an emission wavelength of 461 nm.

A TUNEL assay was performed using an *In Situ* Cell Death Detection Kit (Roche, Germany) according to the manufacturer’s instructions with a few modifications. In brief, 1-week-old *Arabidopsis* roots were fixed in 4% (w/v) paraformaldehyde in PBS (pH 7.4) for 20 min. Then, the fixed seedlings were washed for 30 min with PBS before immersing in permeabilization solution (0.1% Triton X-100, 0.1% sodium citrate) for 2 min. After washing the samples three times with PBS, 50 μL aliquot of TUNEL reaction mixture was then added to the sample, and it was incubated for 1 h (37°C) in a humidified atmosphere in the dark. After being rinsed three times with PBS, photographs were taken under a Leica SP8 laser scanning confocal microscope with an excitation wavelength of 488 nm and an emission wavelength of 530 nm.

### Statistical Analysis

All the experiments were run in triplicate or more unless otherwise indicated, and the results reported in this study are presented as the mean ± SD. Five roots were used for every kind of experiment (ROS assays, GUS assays, and cell death detection). Data were analyzed by a two-tailed Student’s *t*-test using GraphPad Prism 7.0. The significance levels are ^∗^*P* < 0.05, ^∗∗^*P* < 0.01, and ^∗∗∗^*P* < 0.001.

## Results

### Accumulation of ROS Induced by Hypoxia Response of Waterlogged *Arabidopsis thaliana*

To validate the system of waterlogging, we analyzed the relative expression of the hypoxia-responsive genes *ADH1, PDC1, PDC2, SUS1, SUS4, LDH*, hemoglobin 1 (*HB1*), hypoxia-responsive unknown protein 43 (*HUP43*), and LOB domain-containing protein 41 (*LBD41*) (Hunt et al., 2002; Mustroph et al., 2010) in wild-type roots at 0, 4, 8, 12, and 24 h after waterlogging. The expression of the hypoxia-responsive genes *ADH1, PDC1, SUS1, HB1, HUP43*, and *LBD41* started to increase within 4 h and peaked at 4 h (Supplementary Figure S1), and expression of *PDC2, SUS4*, and *LDH* peaked at 8 h under waterlogged conditions (Supplementary Figure S1). These results demonstrate that the system is appropriate to study the hypoxia response of waterlogged *Arabidopsis* roots.

**FIGURE 1:**
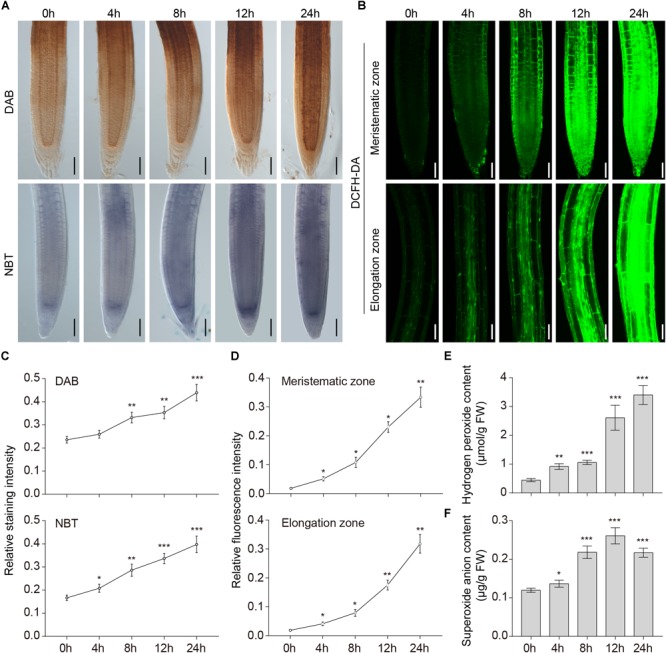
Accumulation of ROS induced by waterlogging. **(A)** DAB staining for H_2_O_2_ and NBT staining for superoxide anion in the primary root of wild-type *Arabidopsis* after waterlogging treatment. Bars = 50 μm. **(B)** DCFH-DA staining for ROS in the primary root of wild-type *Arabidopsis* after waterlogging treatment. Bars = 50 μm. **(C–D)** Relative staining intensities calculated from **(A–B)**. **(E)** H_2_O_2_ and **(F)** superoxide anion contents in wild-type roots after waterlogging treatment. All of the experiments have been repeated at least three times. Data shown are the mean ± SD (*n* = 3). ^∗^*P* < 0.05; ^∗∗^*P* < 0.01; ^∗∗∗^*P* < 0.001 by Student’s *t*-test.

We previously showed that accumulation of ROS in wheat root cells was induced by waterlogging (Xu et al., 2013). To investigate whether ROS are involved in the hypoxic response of waterlogged *A. thaliana*, we investigated the generation of ROS in wild-type roots subjected to waterlogging. DAB, NBT, and DCFH-DA staining were used to detect H_2_O_2_, superoxide anion, and ROS *in situ*. Pronounced accumulation of H_2_O_2_, superoxide anion (Figure 1A), and ROS (Figure 1B) was detected in the meristematic and elongation zones of the primary root under waterlogging conditions. In contrast, plants grown under normal conditions had relatively low ROS levels (Figure 1A–D). We also found that the ROS in the root stele seemed to be higher. To further confirm this, we determined the level of H_2_O_2_ and superoxide anion in wild-type roots under waterlogging conditions. The content of H_2_O_2_ in *Arabidopsis* roots was significantly upregulated within 24 h (Figure 1E). The content of superoxide anion started to increase within 4 h and peaked at 12 h (Figure 1F). These findings suggest that waterlogging induced accumulation of ROS.

RBOHD and RBOHF are the most in-depth studied enzymatic ROS-generating systems, and reports of their participation in various plant processes have increased considerably in recent years. To obtain genetic evidence of the response of ROS to waterlogged conditions, we obtained *rbohd, rbohf*, and *rbohdf* mutants and characterized their responses to waterlogging. The results showed that waterlogging markedly suppressed the growth of all plants. Of note, the double mutants and the two single mutants grew more slowly than the wild type (Supplementary Figure S2A). The root length of *rbohdf* was clearly less than that of wild type, *rbohd* and *rbohf* after exposure to waterlogged conditions (Supplementary Figure S2B). Moreover, we used DAB and NBT staining to detect ROS accumulation in roots of wild type and *rboh* mutants. DAB and NBT staining results showed that the root tips of *rbohd, rbohf*, and *rbohdf* mutants accumulated less H_2_O_2_ and superoxide anion than those of the wild type after waterlogging (Supplementary Figures S2C–E). Together, these results indicate that both *RBOHD* and *RBOHF* are required for waterlogging induced ROS generation.

### Changes in *AOX1a* Expression Levels and Antioxidant Enzymes Activities Under Waterlogged Conditions

**FIGURE 2:**
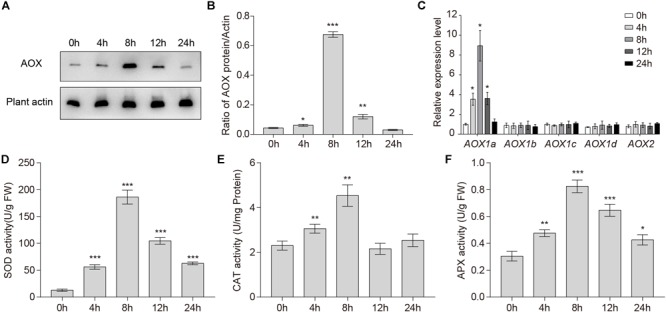
Changes in *AOX1a* expression levels and antioxidant enzymes activities under waterlogged conditions. **(A)** Western blot analysis of the level of total AOX proteins in wild-type roots under waterlogged conditions. Plant actin below the blots to indicate the amount of protein loaded per lane. **(B)** Quantitative analyses of the relative gray values of the western blotting. **(C)** qRT-PCR analyses showing the relative expression of *AOX* genes (*AOX1a, AOX1b, AOX1c, AOX1d*, and *AOX2*) upon waterlogging. **(D)** SOD, **(E)** CAT, and **(F)** APX activities in wild-type roots after waterlogging treatment. All of the experiments were repeated at least three times. Data shown are the mean ± SD (*n* = 3). ^∗^*P* < 0.05; ^∗∗^*P* < 0.01; ^∗∗∗^*P* < 0.001 by Student’s *t*-test.

The fact that AOX can use reductant in excess of either the Cyt pathway capacity or the rate of ATP use suggests that AOX may play an important role in reducing the generation of ROS (Umbach et al., 2005). To test whether waterlogging affected the expression of the AOX protein, total endogenous protein extracted from wild-type roots was immunoblotted with specific monoclonal anti-AOX antibody. The levels of the AOX protein increased upon waterlogging and accumulated to the highest level at 8 h (Figure 2A). As shown in Figure 2B, the gray values of the total AOX protein and the control are significantly different after treatment for 4, 8, and 12 h.

In *A. thaliana*, there are five AOX genes: *AOX1a, AOX1b, AOX1c, AOX1d*, and *AOX2* (Giraud et al., 2008). Because the expression of these five *AOX* genes in response to various stresses is complex, we investigated their transcription levels in wild-type roots after waterlogging. qRT-PCR analyses showed that only the transcription level of *AOX1a* was significantly increased. The transcription level of *AOX1a* started to increase within 4 h, and peaked at 8 h (Figure 2C), which was consistent with the change in the AOX total protein level (Figure 2A). In conclusion, waterlogging may be affecting only the expression of AOX1a.

To counteract oxidative stress in plant cells, three enzymes, SOD, APX, and CAT, are crucial. To further investigate enzymatic mechanisms that scavenge overproduced ROS, we measured the activities of SOD, APX, and CAT in wild-type roots during waterlogging. The activities of SOD, CAT, and APX in *Arabidopsis* roots were significantly upregulated within 24 h (Figure 2D–F). The activities of SOD, CAT, and APX started to increase within 4 h and peaked at 8 h (Figure 2D–F). Together, these findings indicate that activities of antioxidant enzymes were enhanced under waterlogged conditions.

### Waterlogging Induces Formation of Autophagosomes and Autophagy First Occurs in the Root Stele

**FIGURE 3:**
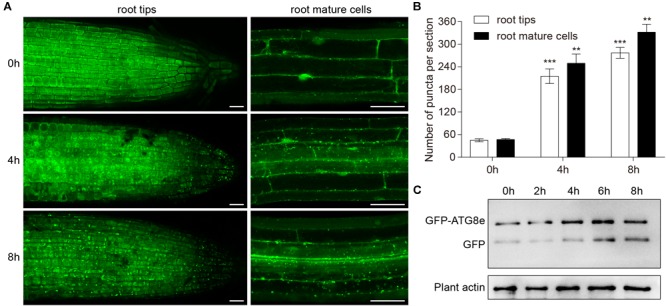
Induction of autophagy by waterlogging. **(A)** *GFP-ATG8e*-labeled dots and ring-like structures accumulate in wild-type roots after waterlogging. Bars = 50 μm. **(B)** Numbers of puncta per root section in the root tips and mature root cells in **(A)**. **(C)** Detection of free GFP generated from transgenic lines expressing *GFP-ATG8e* after waterlogging. Plant actin below the blots to indicate the amount of protein loaded per lane. All of the experiments were repeated at least three times. Data shown are the mean ± SD (*n* = 3). ^∗^*P* < 0.05; ^∗∗^*P* < 0.01; ^∗∗∗^*P* < 0.001 by Student’s *t*-test.

To investigate the role of waterlogging in autophagosome formation in plant cells, we used *Arabidopsis* plants expressing the *GFP-ATG8e* fusion protein as a marker (Contento et al., 2005). Seedlings expressing *GFP-ATG8e* were waterlogged for 4 and 8 h and GFP fluorescence was analyzed using confocal microscopy. In contrast to the root cells of the control, there were more *GFP-ATG8e* labeled dots and ring-like structures in wild-type root tips and mature root cells subjected to waterlogging (Figure 3A,B). At the beginning of autophagy, *GFP-ATG8e* that appeared in the shape of dots formed autophagosomes with other autophagy-associated proteins and subsequently fused with the vacuole. After entering the vacuole, *GFP-ATG8e* was sheared into free GFP and ATG8 hydrolysis fragments by acidic hydrolases. Since the GFP protein is very stable under acidic conditions, it is possible to determine the intensity of autophagy by determining the degradation of *GFP-ATG8e* (Ichimura et al., 2004). Thus, the appearance of free GFP can be used to monitor the extent of autophagy. The waterlogging treatment enhanced the degradation of *GFP-ATG8e*, as measured by the accumulation of free GFP, in comparison to the control (Figure 3C).

**FIGURE 4:**
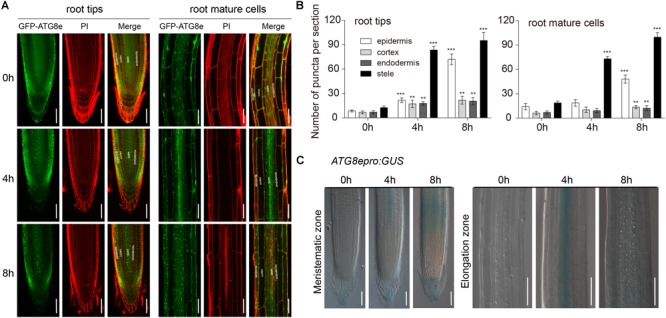
Autophagy occurs first in the root stele. **(A)** Confocal images of primary roots expressing *GFP-ATG8e*. Roots were counterstained with PI. Bars = 50 μm. **(B)** Numbers of puncta per root section in the root tips and mature root cells of wild type in **(A)**. **(C)** The expression of the *ATG8epro:GUS* reporter was monitored in wild-type roots. Bars = 50 μm. All of the experiments have been repeated at least three times. Data shown are the mean ± SD (*n* = 3). ^∗^*P* < 0.05; ^∗∗^*P* < 0.01; ^∗∗∗^*P* < 0.001 by Student’s *t*-test.

To investigate autophagy characteristics upon waterlogging, we examined *ATG8e* expression in *Arabidopsis* roots using seedlings expressing *GFP-ATG8e* and *ATG8epro-GUS*. PI staining was used to distinguish the structure of *Arabidopsis* roots. In contrast to the control, there were more *GFP-ATG8e*-labeled dots and ring-like structures initially observed in the root stele cells after 4 h of waterlogging followed by epidermis of the root after 8 h of waterlogging (Figure 4A). After waterlogging, the appearance of GFP-labeled autophagosome-like structures was significantly increased in the wild-type root stele cells (Figure 4B). GUS staining revealed that the expression of *ATG8e* initially intense in the root stele cells after 4 h of waterlogging followed by other parts of the root after 8 h of waterlogging (Figure 4C). This result shows that waterlogging-activated autophagy initially occurs in the root stele. To further confirm this, we used TEM to examine ultrastructure of *Arabidopsis* root. Analysis of cross sections by TEM revealed autophagy-related structures increased in the root stele after 4 and 8 h of waterlogging treatment and in epidermis of the root after 8 h of waterlogging treatment (Supplementary Figure S3), whereas no obvious difference was detected in other parts of the root (Supplementary Figure S3).

### Loss of AtATG Increases the Plant’s Sensitivity to Waterlogging

Previous studies have implied the function of autophagy under stress conditions (Bassham et al., 2006; Han et al., 2011). To investigate the role of autophagy in the response to waterlogging, we transferred 1-week-old *atg* mutant plants (*atg2, atg5, atg7*, and *atg10*), alongside wild-type plants, to hypoxic conditions induced by waterlogging. We analyzed the *atg* mutants that showed enhanced hypersensitivity to waterlogging, waterlogging + 1% alcohol, and waterlogging + 1% perhydrol (Supplementary Figure S4A). As shown in Supplementary Figure S4B, the primary roots of *atg* mutants were significantly shorter than those of that wild type after waterlogging and the waterlogging + 1% alcohol treatment. During the course of waterlogging + 1% perhydrol stress, most of the wild-type plants survived, but more than half of the *atg* mutants died (Supplementary Figure S4C).

To further evaluate the function of autophagy in the waterlogging response, we transferred 1-week-old wild-type and *atg* mutant seedlings (*atg5* and *atg7*) to waterlogged conditions, and then MDC staining was used to observe autophagosomes. Accumulation of MDC-stained autophagosomes was observed in root tips and mature root cells of wild-type plants subjected to waterlogging (Supplementary Figure S4D), whereas the number of autophagosomes was significantly decreased in the *atg* mutant root cells after waterlogging (Supplementary Figure S4E).

### The Level of Autophagy Decreased in *rboh* Mutants and Accumulation of ROS Increased in *atg* Mutants After Waterlogging

**FIGURE 5:**
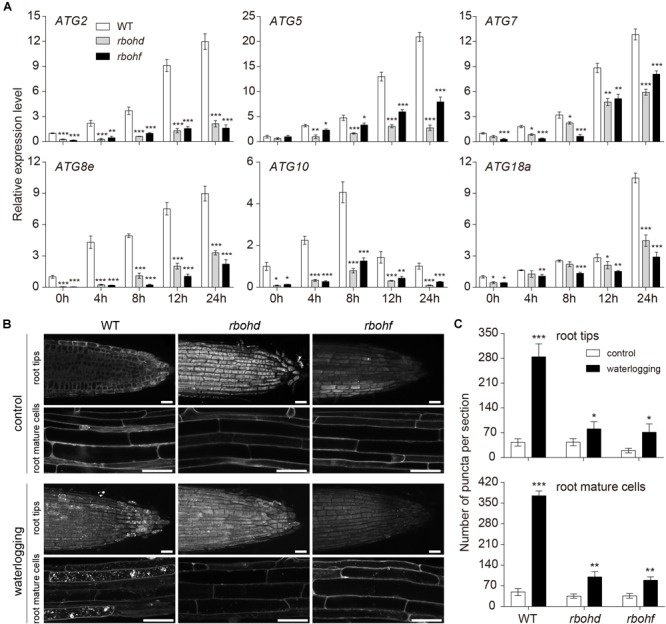
The level of autophagy attenuated in *rboh* mutants after waterlogging. **(A)** Relative transcription levels of autophagy-related genes (*ATG2, ATG5, ATG7, ATG8e, ATG10*, and *ATG18a*) in the wild-type and *rboh* mutants upon waterlogging. **(B)** Confocal microscopy of MDC-stained wild-type, *rbohd*, and *rbohf* seedlings after waterlogging. Bars = 50 μm. **(C)** Quantification of autophagosome number in the root tips and mature root cells of wild-type, *rbohd*, and *rbohf* under waterlogged conditions as in **(B)**. All of the experiments have been repeated at least three times. Data shown are the mean ± SD (*n* = 3). ^∗^*P* < 0.05; ^∗∗^*P* < 0.01; ^∗∗∗^*P* < 0.001 by Student’s *t*-test.

Under waterlogged incubation conditions, accumulation of ROS and autophagy are induced. To investigate the relationship between ROS and autophagy, qRT-PCR was used to quantify the expression levels of *ATG* genes in the wild-type and *rboh* mutants exposed to waterlogging. In comparison to the wild type, the expression of *ATG* genes was significantly downregulated in the *rboh* mutants (Figure 5A). To further confirm the qRT-PCR data, we observed autophagosomes of *rboh* mutants following waterlogging using MDC staining. Upon waterlogging exposure, the appearance of autophagosome-like structures was decreased in the *rbohd* and *rbohf* mutants in comparison with the wild type (Figure 5B,C). We also used TEM to monitor the autophagic activity after waterlogging. The results showed that lower levels of autophagic activity were detected in the *rboh* mutants than in wild type after waterlogging (Supplementary Figures S5A,B). These data demonstrated that the occurrence of autophagy is significantly decreased in *rboh* mutants after waterlogging.

**FIGURE 6:**
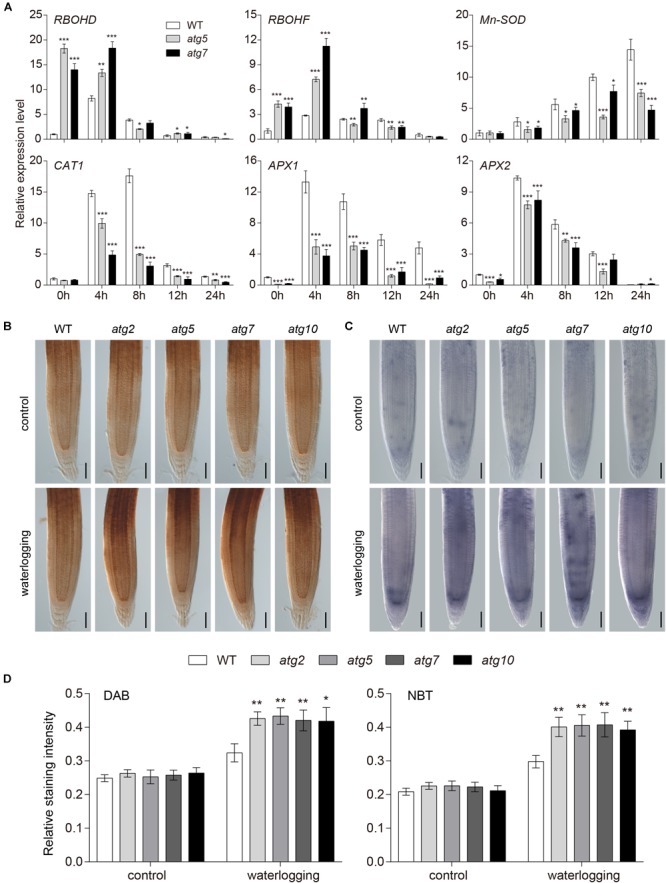
Accumulation of ROS increased in *atg* mutants after waterlogging. **(A)** Relative expression levels of genes encoding enzymes involved in production or reduction of ROS in the wild-type and *atg* mutants after waterlogging treatment. **(B)** DAB staining for H_2_O_2_ and **(C)** NBT staining for superoxide anion in primary root of wild-type and *atg* mutants after waterlogging treatment. Bars = 50 μm. **(D)** Relative staining intensities calculated from **(B–C)**. All of the experiments have been repeated at least three times. Data shown are the mean ± SD (*n* = 3). ^∗^*P* < 0.05; ^∗∗^*P* < 0.01; ^∗∗∗^*P* < 0.001 by Student’s *t*-test.

In order to further investigate the interrelation between ROS and autophagy, we analyzed the mRNA expression level of biosynthesis and scavenging genes of ROS in the wild-type and *atg* mutants upon waterlogging. In comparison to the wild type, the transcription level of *RBOHD* and *RBOHF* was notably increased in the *atg* mutants at 0 and 4 h of waterlogging (Figure 6A). However, the expressions of *SOD, CAT1, APX1*, and *APX2*, which constitute the enzymatic scavenging system to eliminate ROS in plants, were significantly reduced in the *atg* mutants in comparison to the wild type (Figure 6A). In that case, the *atg* mutant plants exhibited excessive accumulation of ROS compared with the wild type under waterlogging stress. In order to further verify higher levels of ROS in *atg* mutants, we transferred 1-week-old *atg* mutant plants, alongside wild-type plants, to DCFH-DA dye. DCFH-DA staining showed higher accumulation of ROS in *atg* mutant plants under waterlogging conditions, in comparison to wild type (Supplementary Figures S6A,B). In comparing the DAB and NBT staining in root tips of the wild-type and *atg* mutants, we found the H_2_O_2_ and superoxide anion levels were both greatly elevated in the *atg* mutants after waterlogging treatment, compared with those of the wild type (Figure 6B–D). Waterlogging treatment significantly enhanced the production of ROS in both wild-type and *atg* mutants (Figure 6B–D and Supplementary Figure S6). In summary, accumulation of ROS is increased in *atg* mutants after waterlogging.

### Effect of Autophagy on PCD During Waterlogging

**FIGURE 7:**
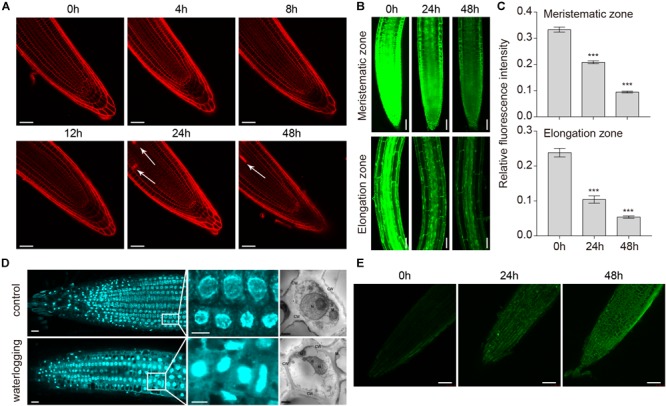
Programmed cell death in roots of *A. thaliana* induced by waterlogging. **(A)** Confocal images of wild-type roots stained with propidium iodide (PI). Arrows indicate the cell death. Bars = 50 μm. **(B)** Confocal images of the detection of cell viability in primary roots. Bars = 50 μm. **(C)** Meristematic and elongation zones of wild type of FDA staining intensity are quantified by Image pro plus 6.0 software. **(D)** Confocal images and TEM images of the detection of nucleus in primary roots with or without waterlogging treatment. CW, cell wall; M, mitochondrion; N, nucleus; G, Golgi body. Bars = 50 μm. **(E)** TUNEL assay of DNA fragmentation (stained in green) in root tip cells of wild-type seedlings. Bars = 50 μm. All of the experiments have been repeated at least three times. Data shown are the mean ± SD (*n* = 3). ^∗^*P* < 0.05; ^∗∗^*P* < 0.01; ^∗∗∗^*P* < 0.001 by Student’s *t*-test.

*Arabidopsis* hypocotyls form lysigenous aerenchyma as a consequence of cell death of secondary xylem during hypoxia (Mühlenbock et al., 2007). To investigate the PCD process upon waterlogging, 1-week-old wild-type seedlings were treated with PI, which cannot pass through a live cell membrane, but can cross a damaged cell membrane and stain the nucleus. PI staining showed that cell death started at 24 h in the primary root, whereas under normal conditions it was hardly detected (Figure 7A). We used FDA staining to show the effects of waterlogging on cell viability in the meristematic and elongation zones of wild-type roots after waterlogging for 24 and 48 h. *Arabidopsis* plants in the control showed strong FDA fluorescence, whereas weak fluorescence was detected under waterlogging conditions (Figure 7B,C). Typical characteristic of PCD is the occurrence of morphological changes in the nucleus and the cleavage of genomic DNA at internucleosomal sites by endogenous nucleases. To further determine whether the root cell death induced by waterlogging is a kind of PCD, we used DAPI staining and TEM to show the effects of waterlogging on the chromatin condensation of wild-type roots. Confocal images and TEM images showed that a marked increase in DAPI fluorescence and condensed and moon-shaped nuclei were detected under waterlogging conditions (Figure 7D). We also investigated the internucleosomal fragmentation of the DNA by TUNEL assay. TUNEL-positive signals were detected in the meristematic zone to the elongation zone from root tips after waterlogging treatment (Figure 7E). These results indicate that waterlogging induced cell death in *Arabidopsis* roots is a kind of PCD.

**FIGURE 8:**
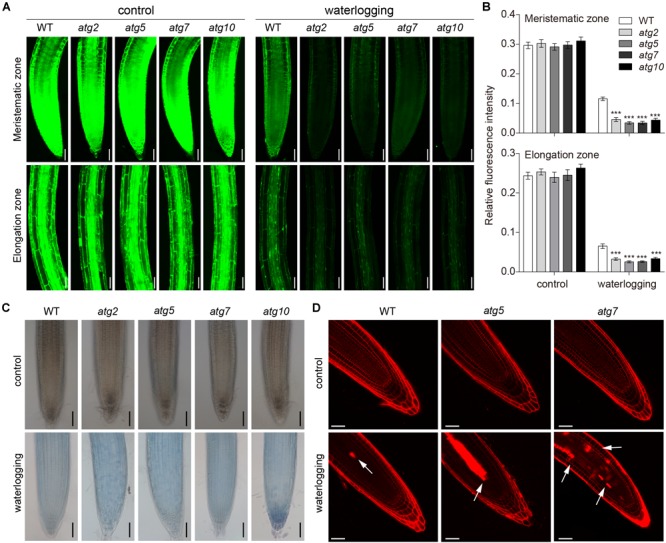
Autophagy regulates the PCD process upon waterlogging. **(A)** Confocal images of the detection of cell viability in primary roots of *atg* mutants in comparison with wild type. Bars = 50 μm. **(B)** Meristematic and elongation zones of wild-type and *atg* mutants of FDA staining intensity are quantified by Image pro plus 6.0 software. **(C)** Trypan blue staining cell death in root of wild-type and *atg* mutants without treatment or waterlogged for 8 h. Bars = 50 μm. **(D)** Cell death of the root of wild-type and *atg* mutants visualized by PI staining. Arrows indicate the cell death. Bars = 50 μm. All of the experiments have been repeated at least three times. Data shown are the mean ± SD (*n* = 3). ^∗^*P* < 0.05; ^∗∗^*P* < 0.01; ^∗∗∗^*P* < 0.001 by Student’s *t*-test.

To examine whether autophagy is also involved in the PCD process in *Arabidopsis* roots, 1-week-old *atg* mutant plants were waterlogged. FDA staining was used to observe cell viability of waterlogged wild-type and *atg* mutants. The fluorescence intensity of *atg* mutants was obviously weaker than that of the wild type in the waterlogged treatment (Figure 8A). However, the fluorescence intensity of wild-type and *atg* mutants grown under normal conditions was fairly similar (Figure 8B). Waterlogging triggered cell death, as indicated by the blue color, in root of the wild-type and *atg* mutants (Figure 8C). In comparison to the wild type and controls, much higher levels of cell death were observed in the roots of *atg* mutants after waterlogging (Figure 8C). To further evaluate the role of autophagy in PCD, we examined cell death in *Arabidopsis* root subjected to waterlogging. In comparison to the wild type, cell death was significantly increased in the *atg* mutants after exposure to waterlogging. And cell death was mainly concentrated in root stele (Figure 8D). In conclusion, autophagy has effect on PCD in *Arabidopsis* roots during waterlogging.

## Discussion

Autophagy, which etymologically means “to eat oneself,” is a highly conserved process that involves vacuolar degradation of damaged or toxic constituents and is induced by ROS accumulation and other factors (Liu et al., 2009). Although autophagy was first described in plants a number of years ago, in the past few years, the study of autophagy molecular mechanisms and regulatory pathways has flourished. In this paper, we demonstrate the effect of waterlogging-induced autophagy on PCD in *Arabidopsis* roots. First, the levels of ROS were found to increase and the activities of SOD, CAT, and APX were significantly increased to cope with ROS under waterlogged conditions (Figure 1, 2). Second, waterlogging-induced autophagy was observed in the stele of *Arabidopsis* roots under waterlogged conditions (Figure 3, 4). Third, *atg* mutant plants displayed enhanced sensitivity to waterlogging stress (Supplementary Figure S4). Fourth, ROS signaling contributed to autophagy, and accumulation of ROS decreased in *Arabidopsis* roots under waterlogged conditions as a result of autophagy (Figure 5, 6 and Supplementary Figures S5, S6). Finally, autophagy-defective mutants showed much higher levels of cell death in comparison to the wild type (Figure 7, 8). Together, this evidence suggests that autophagy is important for *A. thaliana* survival during PCD induced by waterlogging.

In higher plants, ROS are considered to be an important signaling molecules that are produced by different mechanisms through electron transport in chloroplasts and mitochondria or by enzymes in the extracellular space and peroxisomes (Wrzaczek et al., 2013). Plant NADPH oxidase, also known as RBOH, is a major source of ROS in plant–pathogen interactions (Torres and Dangl, 2005). It plays an important role in plant response to abiotic and biological stresses, regulation of plant growth and development, and ROS signal transduction (Gechev et al., 2006; Suzuki et al., 2011). The observations that *rbohd, rbohf*, and *rbohdf* mutants accumulated less H_2_O_2_ and superoxide anion than wild type after waterlogging (Supplementary Figures S2C–E) and waterlogging suppressed the growth of *rbohd, rbohf*, and *rbohdf* mutants (Supplementary Figures S2A,B) are a confirmation of the data previously published (Chen et al., 2015). This suggests that RBOHD and RBOHF are required for ROS accumulation in waterlogged *Arabidopsis* roots. The ROS signaling pathway includes the rapid generation and removal of various forms of ROS to maintain the homeostasis of ROS levels (Mittler et al., 2011). However, the dynamic balance between production and clearance of ROS may be destroyed by certain biotic and abiotic stresses (Apel and Hirt, 2004). Under oxidative stress induced by ROS accumulation, autophagy is involved in reducing oxidative damage and ROS by scavenging damaged organelles, and compared with the wild type, there is a large amount of oxidatively damaged protein accumulation in autophagic mutant plants (Xiong et al., 2007). Consistent with previous observations (Chen et al., 2015), our quantitative PCR analysis showed that the transcription levels of *RBOHD* and *RBOHF* were significantly elevated and antioxidant enzyme genes were significantly decreased in the waterlogged *atg* mutant roots (Figure 6A). Previous studies have shown that *atg* mutants accumulated more H_2_O_2_ than wild type upon submergence (Chen et al., 2015). In our study, NBT and DCFH-DA staining showed higher accumulation of superoxide anion and ROS in *atg* mutant plants under waterlogging conditions, in comparison to wild type (Figure 6B–D and Supplementary Figure S6). While ROS levels were increased in the wild-type and *atg* mutants, there were significant differences among genotypes (Supplementary Figures S4A–C), suggesting that ROS are likely to be scavenged as a result of autophagy under waterlogged conditions. During animal starvation stress, ROS activates autophagy by directly regulating the ATG4 protein (Scherz-Shouval et al., 2007). Autophagy can be induced by different signaling pathways under different abiotic stresses in *Arabidopsis*, such as autophagy induced by starvation and salt stress, which depends on the activity of NADPH oxidase, in contrast to osmotic stress-induced autophagy (Liu et al., 2009). We observed that the expression of *ATG* genes was markedly reduced in the *rboh* mutants in comparison to the wild type (Figure 5A). The waterlogging-induced appearance of autophagosome-like structures was decreased in the *rbohd* and *rbohf* mutants in comparison with the wild type (Figure 5B,C and Supplementary Figure S5). This is consistent with the few autophagosome-like structures that were observed in the *rboh* mutants after submergence (Chen et al., 2015). This suggests that ROS participate in the process of autophagy.

Alternative oxidase helps to remove excess ROS in response to abiotic stress (Umbach et al., 2005). Recent studies have shown that AOX1a-OE plants pretreated with 1-(aminocarbonyl)-1-cyclopropanecarboxylic acid (ACC) exhibited higher whereas *aox1a* showed lower autophagic activity than wild-type plants. ATG8d- and ATG18h-silenced plants had significantly lower levels of AOX protein (Zhu et al., 2018). Applying exogenous H_2_O_2_ and its scavenger DMTU to AOX1a-OE and *aox1a* plants, respectively, found the autophagic activity reduced in AOX1a-OE and increased in *aox1a* plants under drought in response to ACC (Zhu et al., 2018). We found that the expression levels of the AOX protein increased and accumulated to the highest level at 8 h under waterlogging (Figure 2A). Furthermore, it found that only the transcription level of *AOX1a* was significantly increased and it peaked at 8 h (Figure 2B). We also found there were more autophagic structures initially observed in the root stele cells after 4 h of waterlogging followed by epidermis of the root after 8 h of waterlogging (Figure 4A,B). AOX1a-dependent ROS signaling maybe a key factor for the induction of autophagy upon waterlogging. Besides, the activities of antioxidant enzymes started to increase within 4 h and peaked at 8 h (Figure 2D–F). Together, waterlogging may induce autophagy through the regulation of ROS by antioxidant enzymes and AOX1a. All these need further experiments to prove.

Programmed cell death, an autonomous and orderly form of cell death controlled by genes, occurs during plant development or in response to biotic and abiotic stresses (Gadjev et al., 2008). As a signal molecule, ROS can not only promote the process of PCD but also inhibit it (Patel et al., 2006). In recent years, an increasing number of reports suggest that abiotic stresses are associated with the appearance of ROS, which are involved in the induction of autophagy. However, the role of autophagy in PCD induction remains elusive. A dual role of autophagy in plant PCD (autophagy acts as either an initiator or executioner of cell death) has been proposed (Minina et al., 2014). Autophagy plays an essential role in plant innate immunity and negatively regulates PCD (Liu Y. et al., 2005). Previous studies have shown that PCD phenotypes may require the change of SA signaling in the autophagy defective mutants after submergence (Chen et al., 2015). Disruptions of *ATG10* enhance PCD compared to the wild type (Phillips et al., 2008). Enhanced trypan blue staining in *atg5* and *atg7* mutants were observed in UV-B-exposed leaves (Izumi et al., 2017). Compared with previous studies, this study focused only on the root of 1 weeks old *Arabidopsis*. Our findings from trypan blue and PI staining analyses showed that cell death was significantly upregulated in *atg* mutants upon waterlogging (Figure 8C,D). We also used FDA staining to observe the cell viability of waterlogged wild-type and *atg* mutant plants. The fluorescence intensity of the *atg* mutants was obviously weaker than that of the wild type after waterlogging (Figure 8A), whereas that of plants grown under normal conditions was fairly similar (Figure 8B). Furthermore, we found that waterlogging-induced root cell death is a kind of PCD by observing nuclear morphological changes and internucleosomal fragmentation of the DNA (Figure 7D–E). Thus, we propose that PCD may be affected by waterlogging induced autophagy. Most interestingly, this study also found that waterlogging induced ROS generation, autophagy and PCD process mainly occurred in the root stele. Recent studies have shown that the expression of HRE2 which is a member of oxygen-sensing subgroup VII AP2/ERFs was increased in the root stele at hypoxic conditions (Eysholdt-Derzsó and Sauter, 2017). It is probably due to oxygen-sensing subgroup VII AP2/ERFs mainly expressed in stele, leading to ROS generation, autophagy, and PCD process mainly occurred in the root stele.

**FIGURE 9:**
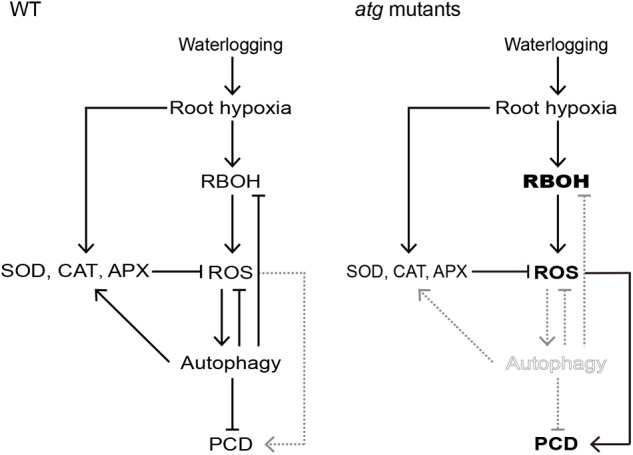
Proposed working model for signaling pathways that regulate PCD in *Arabidopsis* roots. In waterlogged wild-type roots, hypoxia induces both *RBOH* expression (leading to an increase in ROS), as well as increasing the expression and activities of ROS-scavenging enzymes CAT, APX, and SOD. The RBOH-dependent increase in ROS activates autophagy which alleviates PCD. However, autophagy deficiency of the *atg* mutants disrupts the induction of antioxidant enzymes expression and the inhibition of RBOH expression by autophagy. In that case, excessive accumulation of ROS contributes to PCD in *atg* mutant roots.

Based on the results presented above, a model for effect of waterlogging-induced autophagy on PCD process in *Arabidopsis* roots can be proposed (Figure 9). In waterlogged wild-type roots, continued respiration and constrained gas influx can rapidly induce hypoxia. During the PCD process in waterlogged *Arabidopsis* roots, the amount of ROS is increased by enhancement of *RBOH*. In addition, the activities of SOD, APX, and CAT are upregulated to alleviate the generation of ROS under waterlogged conditions. ROS results in a significant increase in the transcription level of *ATG* genes and the formation of autophagosomes and autophagy, which in turn reduces the levels of ROS. Autophagy may induce the expression of antioxidant enzymes and inhibit the expression of *RBOH*. Finally, autophagy alleviates PCD in *Arabidopsis* roots. By contrast, autophagy deficiency of the *atg* mutants disrupts the induction of antioxidant enzymes expression and the inhibition of *RBOH* expression by autophagy. Autophagy deficiency also disrupts the interaction between ROS and autophagy, which may alleviate PCD. In that case, excessive accumulation of ROS increased PCD in *atg* mutant roots.

## Author Contributions

BG and ZZ conceived and designed the experiments, and revised the manuscript. BG performed the experiments and wrote the manuscript. ZL and DL performed the part of experiments. All authors discussed the results and revised the manuscript.

## Conflict of Interest Statement

The authors declare that the research was conducted in the absence of any commercial or financial relationships that could be construed as a potential conflict of interest.

## Footnotes

1 http://www.adobe.com
