# Supplementary material for: Effect of Waterlogging-Induced Autophagy on Programmed Cell Death in Arabidopsis Roots
Source: Front Plant Sci. 2019 Apr 11;10:468. doi: 10.3389/fpls.2019.00468 (PMC6470631; doi:10.3389/fpls.2019.00468)
Supplement: Supplementary file 1 [file Table_1.DOCX]

**Title:** Effect of waterlogging-induced autophagy on programmed cell death in *Arabidopsis* roots

**Authors:** Bin Guan^1^, Ze Lin^1^, Dongcheng Liu^1^, Chengyang Li^1^, Zhuqing Zhou^1, *^, Fangzhu Mei^2^, Jiwei Li^3^, Xiangyi Deng^3^

**Table S1.** Information of genes included in this study.

| **Gene** | **Locus ID** | **Full name** | **Products** |
| --- | --- | --- | --- |
| *ADH1* | AT1G77120 | alcohol dehydrogenase 1 | alcohol dehydrogenase |
| *PDC1* | AT4G33070 | pyruvate decarboxylase 1 | pyruvate decarboxylase |
| *PDC2* | AT5G54960 | pyruvate decarboxylase 2 | pyruvate decarboxylase |
| *HB1* | AT2G16060 | hemoglobin 1 | class 1 nonsymbiotic hemoglobin |
| *SUS1* | AT5G20830 | sucrose synthase 1 | sucrose synthase |
| *SUS4* | AT3G43190 | sucrose synthase 4 | sucrose synthase |
| *HUP43* | At5G39890 | hypoxia-responsive unknown protein 43 | unknown protein |
| *LBD41* | At3G02550 | LOB domain-containing protein 41 | LOB domain-containing protein |
| *LDH* | AF043130 | lactate dehydrogenase 1 | lactate dehydrogenase |
| *RBOHD* | AT5G47910 | respiratory burst oxidase homolog D | respiratory burst oxidase |
| *RBOHF* | AT1G64060 | respiratory burst oxidase homolog F | respiratory burst oxidase |
| *CAT1* | AT1G20630 | catalase 1 | catalase |
| *APX1* | AT1G07890 | ascorbate peroxidase 1 | ascorbate peroxidase |
| *APX2* | [AT3G09640](https://www.araport.org/locus/AT3G09640) | ascorbate peroxidase 2 | ascorbate peroxidase |
| *Mn-SOD* | [AT3G10920](https://www.araport.org/locus/AT3G10920) | manganese superoxide dismutase | superoxide dismutase |
| *ATG2* | AT3G19190 | autophagy-related 2 | autophagy-related protein |
| *ATG5* | AT5G17290 | autophagy-related 5 | autophagy-related protein |
| *ATG7* | AT5G45900 | autophagy-related 7 | autophagy-related protein |
| *ATG8e* | AT2G45170 | autophagy-related 8e | autophagy-related protein |
| *ATG10* | AT3G07525 | autophagy-related 10 | autophagy-related protein |
| *ATG18a* | AT3G62770 | autophagy-related 18a | autophagy-related protein |
| *AOX1a* | AT3G22370 | alternative oxidase 1a | alternative oxidase |
| *AOX1b* | AT3G22360 | alternative oxidase 1b | alternative oxidase |
| *AOX1c* | AT3G27620 | alternative oxidase 1c | alternative oxidase |
| *AOX1d* | AT1G32350 | alternative oxidase 1d | alternative oxidase |
| *AOX2* | AT5G64210 | alternative oxidase 2 | alternative oxidase |

**Table S2.** Sequence of primers for several genes in RT-qPCR.

| **Gene** | **Name** | **Sequence (5’→3’)** |
| --- | --- | --- |
| *ADH1* | ADH FP | TATTCGATGCAAAGCTGCTGTG |
|  | ADH RP | CGAACTTCGTGTTTCTGCGGT |
| *PDC1* | PDC1 FP | CGATTATGGCACTAACCGGATT |
|  | PDC1 RP | TGTTCACCACCGCCTGATAAC |
| *PDC2* | PDC2 FP | GATTTGGTAGTGTCTTCACCGTTCTATG |
|  | PDC2 RP | TCCTCAAGGGGACACACATTTTTT |
| *HB1* | HB1 FP | TTTGAGGTGGCCAAGTATGCA |
|  | HB1 RP | TGATCATAAGCCTGACCCCAA |
| *HUP43* | HUP43 FP | CTTCGAGCCGTTTTGGATGA |
|  | HUP43 RP | ACGTCACTAACGGAGATCGTCC |
| *LBD41* | LBD41 FP | TGAAGCGCAAGCTAACGCA |
|  | LBD41 RP | ATCCCAGGACGAAGGTGATTG |
| *SUS1* | SUS1 FP | ACGCTGAACGTATGATAACGCG |
|  | SUS1 RP | AACCCTGGAAAGCAAGGCAAG |
| *SUS4* | SUS4 FP | GAAGAGTGAAAGAAGAAGATTTTCTACATTC |
|  | SUS4 RP | TAAAAAACAATGGTGAAGCATATCATAAAA |
| *LDH* | LDH FP | AGGAATGGTGTGGTTGCTGTGAC |
|  | LDH RP | GCAGCTTCTCAGCCTCTTCATCA |
| *ATG2* | ATG2 FP | AATGGATAGCAAGTGGAAGC |
|  | ATG2 RP | AGATAGACCTACCGTTAGCC |
| *ATG5* | ATG5 FP | ACTGATACCATGTGAAGGAG |
|  | ATG5 RP | GTATAGGCATCAAGATCACC |
| *ATG7* | ATG7 FP | GAAGATTGTCTAGGTCGTGG |
|  | ATG7 RP | CCTGCTTTCTCTTGTATCGG |
| *ATG8e* | ATG8e FP | CTGAAGCTGGAAGGATCAGG |
|  | ATG8e RP | GCTTGACATTAGCTCTCCTG |
| *ATG10* | ATG10 FP | ATCATACAAGGTTCCTGTGC |
|  | ATG10 RP | GATGTAGCTTGAACCATGGC |
| *ATG18a* | ATG18a FP | AGATCATGCTTGCTTCGCTG |
|  | ATG18a FP | AGAGTTCTCCGATACATCGG |
| *RBOHD* | RBOHD FP | ATTACAAGCACCAAACCAG |
|  | RBOHD RP | TGCCAAGCCATAACATCA |
| *RBOHF* | RBOHF FP | CTGCGGTTTCGCCATTC |
|  | RBOHF RP | TGTTTCGTCGGCTCTG |
| *CAT1* | CAT1 FP | CCAAGTCTCACATTCAGGAG |
|  | CAT1 RP | TCTGATAGCTTCCTCATCCG |
| *APX1* | APX1 FP | TGTCAATCAAGGACTGGAGG |
|  | APX1 RP | CAAATGGTCACAACCCTTGG |
| *APX2* | APX2 FP | AATATGCTGCAGATGAGGATGC |
|  | APX2 RP | CAAGAATCAAGGAGGTAGGAGATG |
| *Mn-SOD* | Mn-SOD FP | ACAAGTTTGTACAAAAAAGCAGGCT |
|  | Mn-SOD RP | ACCACTTTGTACAAGAAA GCTGGGT |
| *AOX1a* | AOX1a FP | ATGATGATAACTCGCGGTGGAGC |
|  | AOX1a RP | GCAACATTCAAAGAAAGCCGAATC |
| *AOX1b* | AOX1b FP | ATGATGATGAGTCGTCGCTATG |
|  | AOX1b RP | CCCATTAAAGCCCATTTAGG |
| *AOX1c* | AOX1c FP | CAAATCTCCCTTGAATCCG |
|  | AOX1c RP | GCTCTTCTGATTCAGTGATATCC |
| *AOX1d* | AOX1d FP | CCCAACTGTTGTTACTCATG |
|  | AOX1d RP | CACAGCTTTGTGACTTTGTC |
| *AOX2* | AOX2 FP | ATGGGTATGAGTTCTGCATCG |
|  | AOX2 RP | TTAGTGATAACCAATCGGAGCTG |
| *ACTIN2* | Actin FP | TGGCGATGAAGCTCAATCC |
|  | Actin RP | CACTGGCATAAAGAGAAAG |


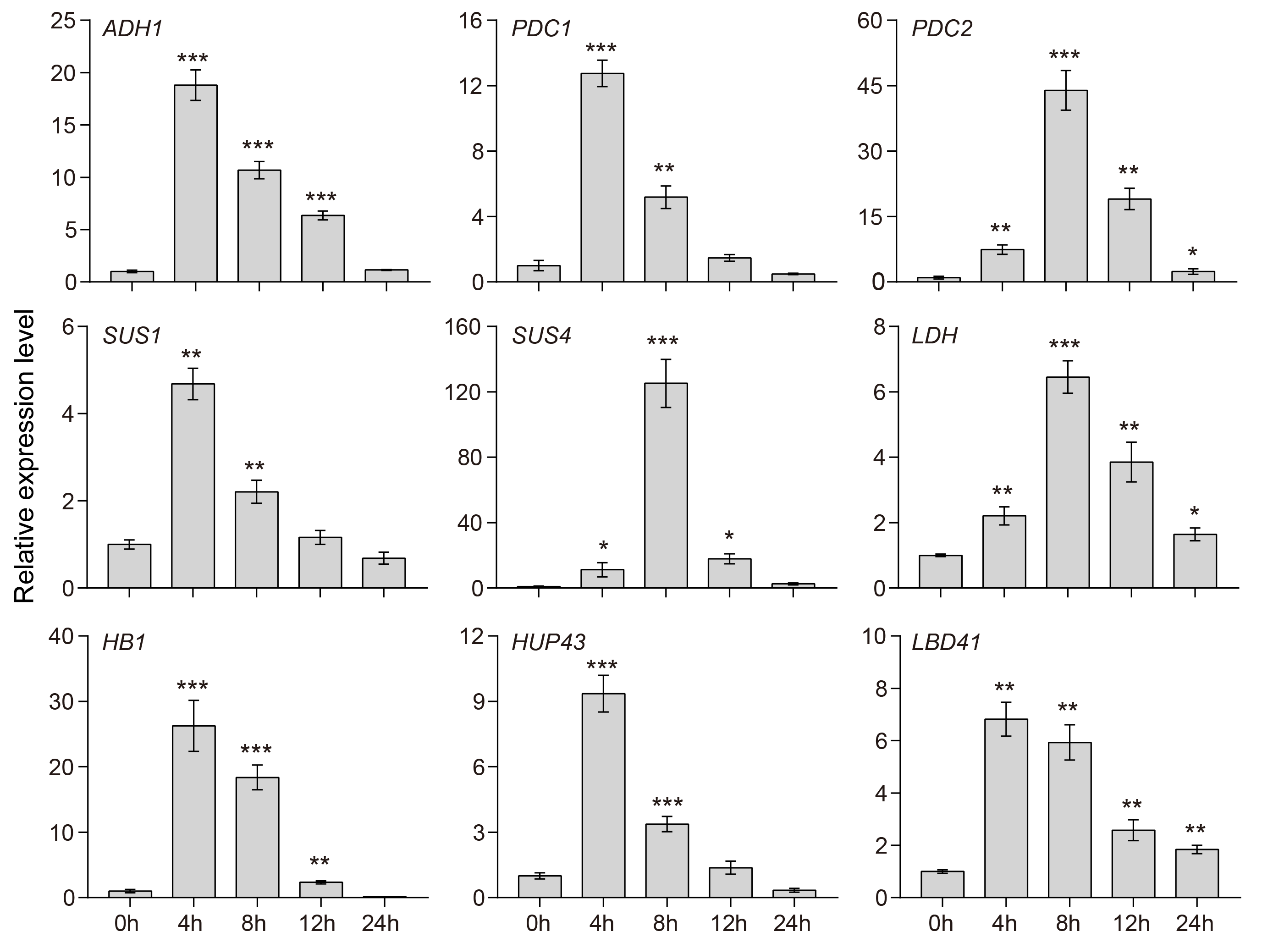


**Figure S1.** Relative transcription levels of hypoxia-responsive genes (*ADH1, PDC1, PDC2, SUS1, SUS4, LDH, HB1, HUP43,* and *LBD41*) in wild type roots upon waterlogging. The experiments have been repeated at least 3 times. Data shown are the mean ± SD (n = 3). *, P < 0.05; **, P < 0.01; ***, P < 0.001 by the Student’s *t*-test.

**
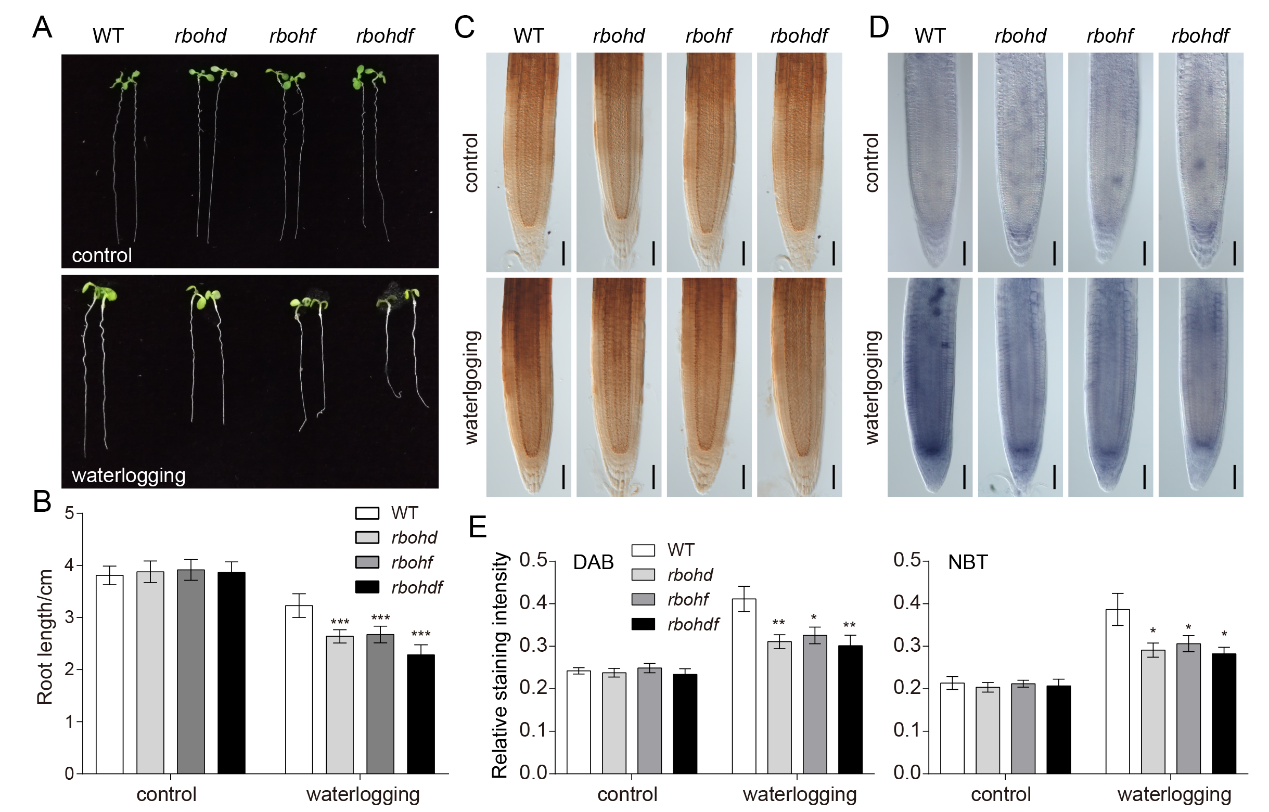
**

**Figure S2.** *RBOHD* and *RBOHF* are required for waterlogging induced ROS generation. (A) Images of one-week-old wild type and *rboh* mutants (*rbohd*, *rbohf*, and *rbohdf*) after waterlogging treatment for 2d. (B) Root length of WT and *rboh* mutants after waterlogging treatment for 2d. (C) DAB staining for H_2_O_2_ and (D) NBT staining for superoxide anion in primary root of wild type and *rboh* mutants after waterlogging treatment. Bars = 50 μm. (E) Relative staining intensities calculated from (C-D). All of the experiments have been repeated at least 3 times. Data shown are the mean ± SD (n = 3). *, P < 0.05; **, P < 0.01; ***, P<0.001 by the Student’s *t*-test.

**
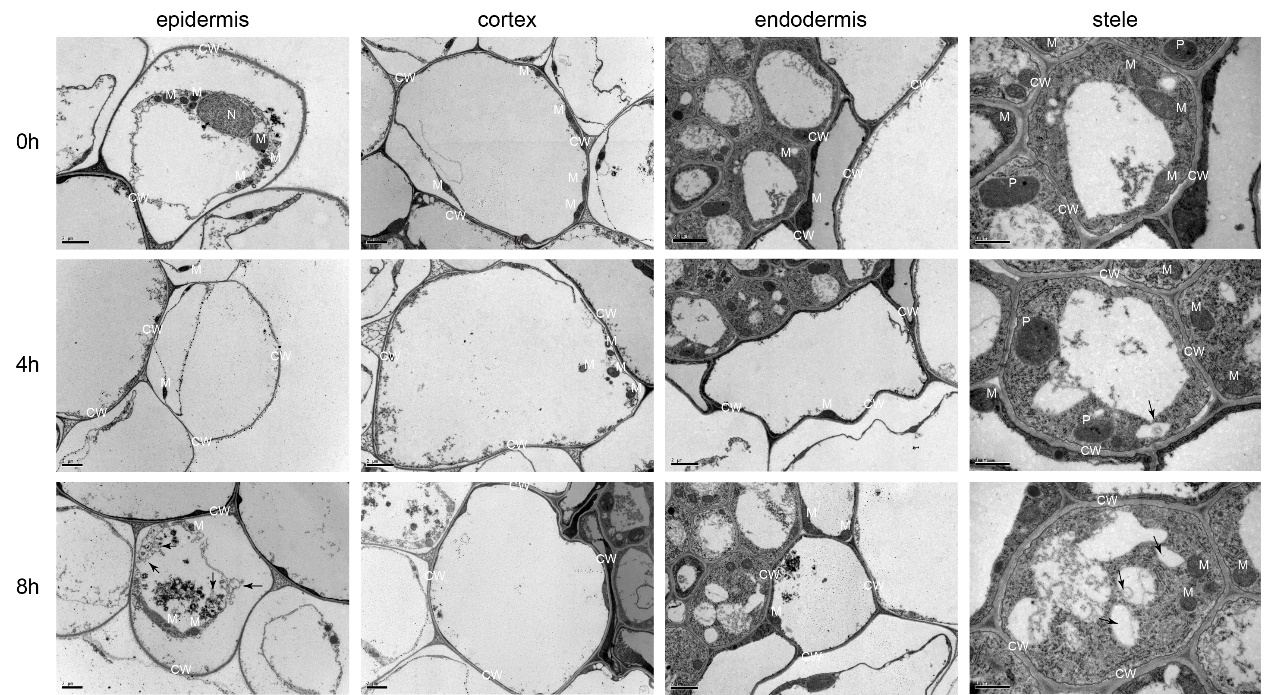
**

**Figure S3.** Representative TEM images in wild type roots after waterlogging treatment. Arrows indicate the autophagosome or autophagic bodies. *CW* cell wall, *M* mitochondrion, *P* peroxisome. Bars = 2 μm.


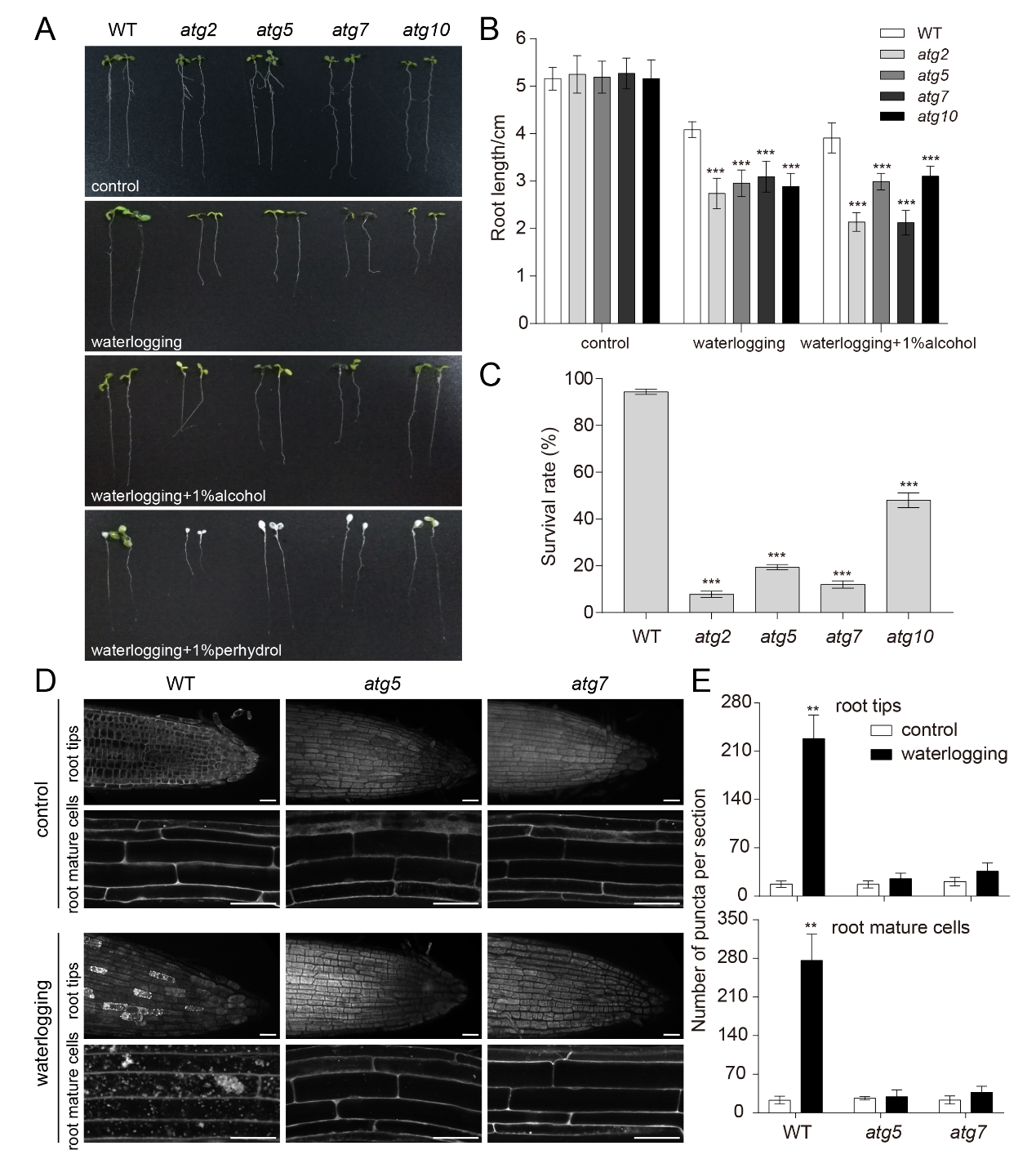


**Figure S4.** *Atg* mutants show enhanced sensitivity to waterlogging. (A) Images of one-week-old wild type and *atg* mutants germinated on MS medium without treatment, after waterlogging treatment for 2 d, after waterlogging + 1% alcohol treatment for 2 d, and after waterlogging + 1% perhydrol treatment for 2 d. (B) Root length of wild type and *atg* mutants germinated on MS medium without treatment, after waterlogging treatment for 2 d, and after waterlogging + 1% alcohol treatment for 2 d. (C) Survival rates of wild type and *atg* mutants after waterlogging + 1% perhydrol treatment for 2 d. (D) Representative confocal images of MDC-stained wild type, *atg5,* and *atg7* seedlings after waterlogging. Bars = 50 μm. (E) Numbers of puncta per root section in the root tips and mature root cells of wild type, *atg5*, and *atg7* in (D). All of the experiments have been repeated at least 3 times. Data shown are the mean ± SD (n = 3). *, P < 0.05; **, P < 0.01; ***, P < 0.001 by Student’s *t*-test.


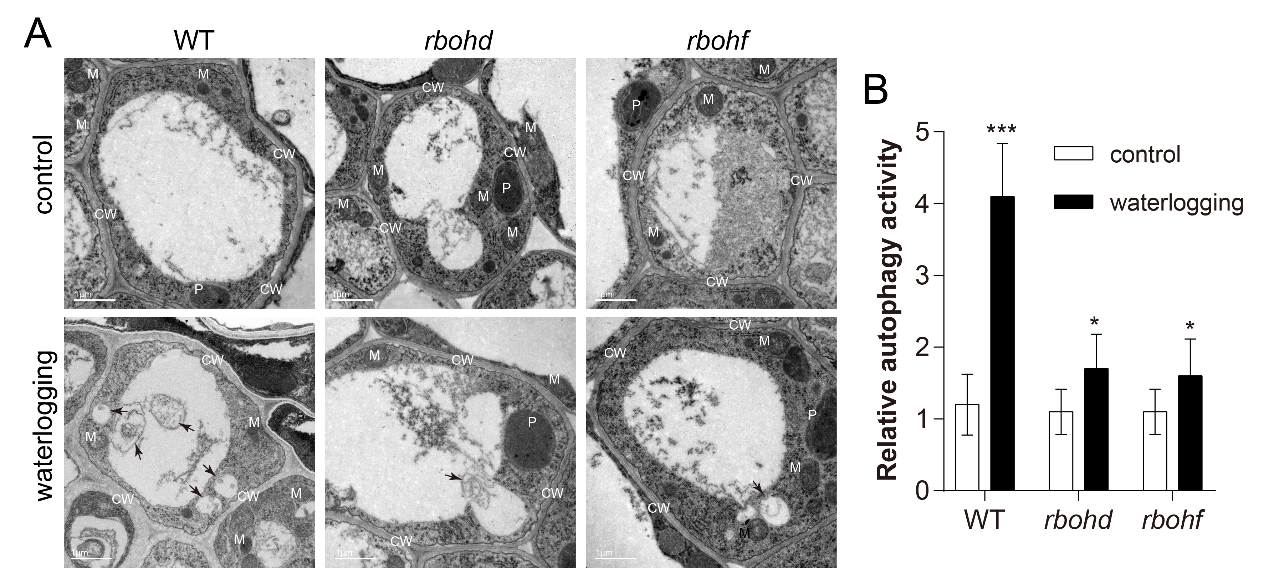


**Figure S5.** Visualizing accumulation of autophagosomes under waterlogging by TEM. (A) Representative TEM images of autophagic structures in waterlogged wild-type, *rbohd*, and *rbohf* seedlings. Arrows indicate the autophagosome or autophagic bodies. *CW* cell wall, *M* mitochondrion, *P* peroxisome. Bars = 1 μm. (B) Relative autophagic activity normalized to activity of WT or *rboh* mutants shown in (A). All of the experiments were repeated at least 3 times. Data shown are the mean ± SD (n = 3). *, P < 0.05; **, P < 0.01; ***, P < 0.001 by the Student’s *t*-test.


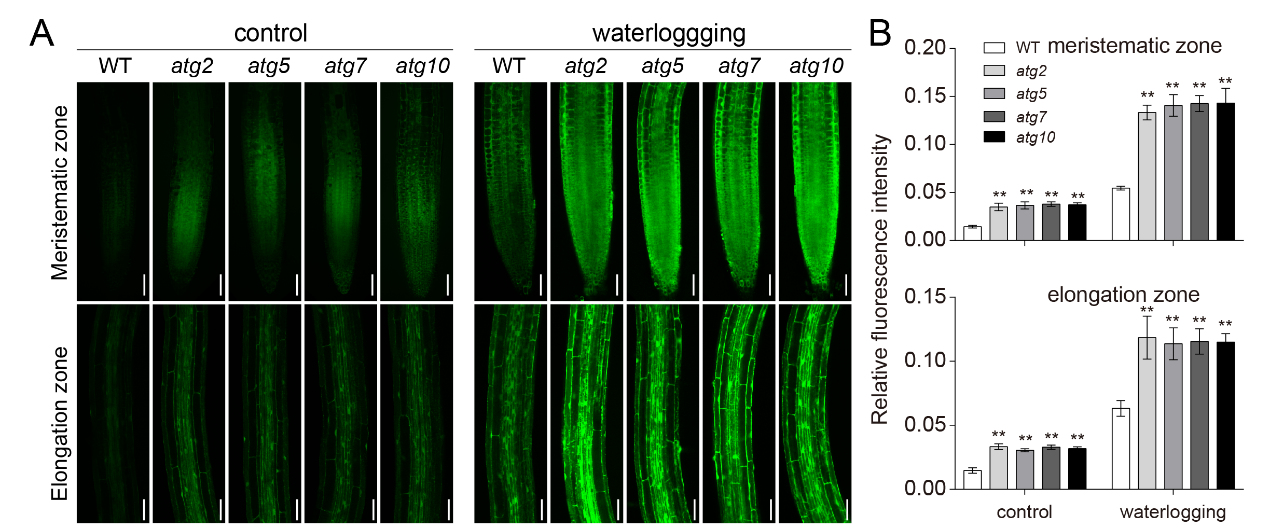


**Figure S6.** Accumulation of ROS increased in *atg* mutants after waterlogging. (A) Representative photographs of the detection of endogenous ROS levels in primary root of *atg* mutants (*atg2, atg5, atg7,* and *atg10*) compared with wild type. Bars = 50 μm. (B) Meristematic and elongation zones of the wild type and *atg* mutants of DCFH-DA staining intensity as determined with Image pro plus 6.0 software. All of the experiments have been repeated at least 3 times. Data shown are the mean ± SD (n = 3). *, P < 0.05; **, P < 0.01; ***, P < 0.001 by the Student’s *t*-test.
